# Effect of thoracic cage width on surgery time and postoperative outcome in minimally invasive esophagectomy

**DOI:** 10.1007/s00464-023-10340-2

**Published:** 2023-09-07

**Authors:** C. Mann, T. Jezycki, F. Berlth, E. Hadzijusufovic, E. Uzun, A. Mähringer-Kunz, H. Lang, R. Klöckner, P. P. Grimminger

**Affiliations:** 1 Department of General-, Visceral- and Transplantation Surgery, University Medical Center Mainz, Mainz, Germany; 2 Department of Diagnostic and Interventional Radiology, University Medical Centre of the Johannes Gutenberg-University Mainz, Mainz, Germany; 3 Department for Interventional Radiology, University Medical Center Lübeck, Lübeck, Germany

**Keywords:** Esophageal cancer, Minimally invasive esophagectomy, Thoracic cage width, Surgical difficulty, MIE, RAMIE

## Abstract

**Introduction:**

Minimally invasive esophagectomy (MIE) for esophageal cancer is a complex procedure that reduces postoperative morbidity in comparison to open approach. In this study, thoracic cage width as a factor to predict surgical difficulty in MIE was evaluated.

**Methods:**

All patients of our institution receiving either total MIE or robotic-assisted MIE (RAMIE) with intrathoracic anastomosis between February 2016 and April 2021 for esophageal cancer were included in this study. Right unilateral thoracic cage width on the level of vena azygos crossing the esophagus was measured by the horizontal distance between the esophagus and parietal pleura on preoperative computer tomography. Patients’ data as well as operative and postoperative details were collected in a prospective database. Correlation between thoracic cage width with duration of the thoracic procedure and postoperative complication rates was analyzed.

**Results:**

Overall, 313 patients were eligible for this study. Thoracic width on vena azygos level ranged from 85 to 149 mm with a mean of 116.5 mm. In univariate analysis, a small thoracic width significantly correlated with longer duration of the thoracic procedure (*p* = 0.014). In multivariate analysis, small thoracic width and neoadjuvant therapy were identified as independent factors for long duration of the thoracic procedure (*p* = 0.006). Regarding postoperative complications, thoracic cage width was a significant risk factor for occurrence of postoperative pneumonia in the multivariate analysis (*p* = 0.045). Dividing the cohort into two groups of patients with narrow (≤ 107 mm, 19.5%) and wide thoraces (≥ 108 mm, 80.5%), the thoracic procedure was significantly prolonged by 17 min (204 min vs. 221 min, *p* = 0.014).

**Conclusion:**

A small thoracic cage width is significantly correlated with longer operation time during thoracic phase of a MIE in Europe, which suggests increased surgical difficulty. Patients with small thoracic cage width may preferably be operated by MIE-experienced surgeons.

**Graphical abstract:**

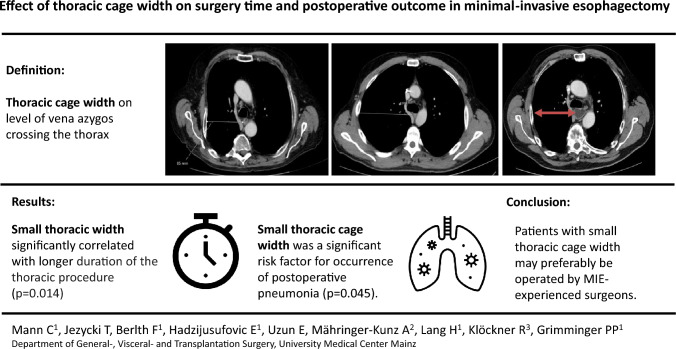

Esophageal cancer remains a global oncological burden with over 500,000 deaths in 2018 [1]. Lately, multimodal therapy strategies have proven to be most effective in reducing its morbidity and mortality. Radical esophagectomy plays the crucial role in curative treatment. However, radical esophageal resection, accessing two visceral cavities, is a complex procedure with overall morbidity up to 59% in Low’s Benchmark of complication after esophagectomy from 2019 [2]. In order to reduce operative trauma, minimally invasive surgery has been introduced in esophageal surgery by Cuschieri et al. [3]. After a period of proofing feasibility and safety of total minimally invasive esophagectomy (MIE), some multi-center randomized trials (beginning with TIME trial) reported significantly lower intraoperative blood loss, lower complication rates as well as shorter duration of postoperative hospital stay in MIE compared to open esophagectomy (OE) [4–7]. However, so far MIE as well as robotic-assisted MIE (RAMIE) is accompanied with longer operation time and flatter learning curves compared to open esophagectomy due to surgical difficulty. In our experience, especially narrow thoraces with small working angles and rigid instruments firstly limit the surgeons’ flexibility around delicate structures and secondly can hamper the effortless creation of the anastomosis in the thorax. In this study, we evaluated the impact of narrow thoracic cavities on procedure duration and postoperative complication rates of MIE as well as RAMIE for esophageal cancer in a Western Europe cohort.

## Methods

The study was conducted according to the guidelines of the Declaration of Helsinki 1975 and approved by the Ethics Committee of the state of Rhineland-Palatinate. Total MIE was introduced in our institution in 2016, RAMIE in 2017. We collected data from all patients operated in our center for esophageal cancer regarding operative details, postoperative complications, and survival in a prospective database. From February 2016 until April 2021, we retrospectively identified 344 patients receiving minimally invasive esophagectomy. We included all 332 patients receiving an Ivor Lewis operation with intrathoracic anastomosis. Due to lacking data regarding OR time or preoperative imaging, 19 patients were excluded from the study. All remaining 313 patients included in the study had received preoperative endoscopy, as well as preoperative computer tomography (CT scan) and routine blood examination. Postoperative complications were defined as any complication classified by Clavien–Dindo (1–5) [8]. These included anastomotic leakage, pleural effusion, pneumonia, postoperative bleeding, chylothorax, recurrent nerve paralysis, and cardiac complications. Severe complications were defined as any adverse event beyond Clavien–Dindo 3b. We evaluated operation time in the thorax as well as postoperative complications rates dependent on thoracic cage width.

### Surgery

Ivor Lewis esophagectomy was performed as a total minimally invasive approach conducted in two stages. In the first stage with supine position of the patient, laparoscopic gastric mobilization, lymphadenectomy at the lesser curvature, and creation of the gastric conduit were performed. In the second stage, the patient was placed to the left semiprone position for maintaining possibility for easy conversion and offering ideal access to the dorsal mediastinum. Thoracoscopically, the esophagus was mobilized and the lymph nodes from the paraesophageal, pulmonary ligament, subcarinal as well as aortopulmonary station were dissected and removed preferable en bloc with the specimen. Port placement is standardized in our institution: in MIE a 12 mm trocar was placed in the fourth intercostal space (ICS) anterior to the scapular margin along the posterior axillary line; two 12 mm trocars were placed along the anterior axillary line on the sixth and eighth ICS and another 12 mm trocar in the 10th ICS [9]. In RAMIE, 8 mm trocars were placed in the fourth, sixth, and 10th intercostal space [ICS], and two 12 mm trocars in the eighth and fifth ICS (assistant trocar) [10]. An esophagogastric anastomosis was created intrathoracically with a circular end-to-end anastomotic stapler through a mini-thoracotomy. In 63.3% of the cases robotic assistance (DaVinci Xi, Intuitive Surgical, Sunnyvale, USA) was used. The surgical steps of the four-arm robotic approach are basically identical to MIE procedure [10]. All surgeries were performed or observed by the same surgeon.

### Measurement of thoracic width

**Fig. 1:**
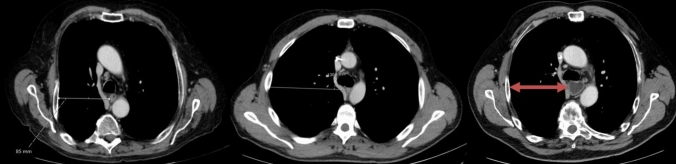
Examples of thoraces with small and wide thoracic width. Measurement of thoracic width on level of vena azygos crossing the esophagus

In our opinion, the most crucial location to measure thoracic cage width for predicting the surgery’s difficulty is on the level of vena azygos crossing the esophagus and the thorax sagitally. Reaching this area in small thoraces and working with limited liberty of action due to small angles of the trocars can hinder a smooth and successful operation. Therefore, we defined the thoracic width as horizontal distance between right thoracic wall and middle of the esophagus on the level where the azygos vein crossed the esophagus sagittally (Fig. 1). This distance was measured in preoperative CT scans of all patients by the same radiologist.

As the relative esophageal location has an additional potential impact on procedure time and complication rates, esophageal location was defined according to a study publishes by Yoshida et al. [11].

### Statistical analysis

Data were reported as median or mean ± SD. All data were tested for normal distribution using the Shapiro–Wilk test. For correlation between continuous variables and operation time, respectively, postoperative complications simple linear or logistic regression was used. Comparing the median or mean either Mann–Whitney *U* Test or student’s *T*-test was used. For correlation between categorical variables and operation time Mann–Whitney *U* test was used. Chi-Square Test was used to test the differences of categorical variables between groups of narrow and wide thoraces. Statistical analysis was performed using standardized biomedical software (SPSS Version 29, IBM Corp, Armonk, NY, USA). Differences were considered significant at *p* < 0.05.

## Results

Demographics, disease, and intraoperative characteristics are demonstrated in Table 1. We included 59 female and 254 male patients with a median age of 65 years. Thoracic width on vena azygos level ranged from 85 to 149 mm with a mean of 116.5 mm. 115 patients received a MIE, 198 patients were operated with robotic assistance. Only 9 patients had a completely deep seated esophagus as defined in the study of Yoshida et al. However, 164 patients in our cohort had a partially left-sided esophagus located on the left side of the trachea in the upper mediastinum. Interestingly, those patients had significantly more often a wide thorax (*p* < 0.001).

**Table 1:** Patient and tumor characteristics, comparison between narrow and wide thorax groups

	Value All patients	Narrow thorax $\le$ 107 mm	Wide thorax $\ge$ 108 mm	*p*-value
Patient characteristics				
Total number	313	60 (19.2)	253 (80.8)	
Sex *n* (%)				< 0.001
Female	59 (18.8)	25 (41.7)	34 (13.4)	
Male	254 (81.2)	35 (58.3)	219 (86.6)	
Age (years–SD)	65 (25–85)	64 (31–85)	65 (25–85)	0.710
Body mass index (kg/m^2^, range)	25.6 (13.8–47)	25.2 (6.2)	25.9 (4.7)	0.354
Thoracic width (mm–SD; range)	116.5 (11; 85–149)			
ASA score *n* (%)				0.690
1	1 (0.3)	0	1 (0.4)	
2	137 (43.8)	25 (41.7)	112 (44.3)	
3	158 (50.5)	30 (50.0)	128 (50.6)	
4	17 (5.4)	5 (8.3)	12 (4.7)	
Charlson comorbidity index				0.104
1–2	12 (3.8)	2 (3.3)	10 (4.0)	
3–4	124 (39.6)	31 (51.7)	93 (36.8)	
> 5	177 (56.6)	27 (45)	150 (59.3)	
Pulmonary comorbidity *n* (%)	67 (19.8)	12 (20.6)	50 (19.8)	0.967
Cardiovascular comorbidity *n* (%)	180 (57.5)	35 (58.3)	145 (57.3)	0.886
Diabetes mellitus *n* (%)	43 (13.7)	7 (11.7)	36 (14.2)	0.604
Left-sided esophagus *n* (%)	164 (52.4)	19 (31.7)	145 (57.3)	< 0.001
Disease characteristics				
Histology *n* (%)				0.254
Squamous cell carcinoma	82 (26.2)	20 (33.3)	62 (24.5)	
Adenocarcinoma	227 (72.5)	40 (66.7)	187 (73.9)	
Other	4 (1.3)	0	4 (1.6)	
Clinical T status *n* (%)				0.146
cT1–2	77 (23.6)	11 (18.3)	66 (26.1)	
cT3–4	229 (73.2)	46 (76.7)	183 (72.3)	
cTx	7 (2.2)	3 (5.0)	4 (1.6)	
Clinical N status *n* (%)				0.308
cN0	81 (25.9)	11 (18.3)	70 (27.7)	
cN+	166 (53)	34 (56.7)	132 (52.2)	
cNx	66 (21.1)	15 (25)	51 (20.2)	
Tumor location *n* (%)				0.860
AEG 1	154 (49.2)	30 (50)	124 (49)	
AEG 2	121 (38.7)	21 (35)	100 (39.5)	
Cervical	1 (0.3)	0 (0)	1 (0.4)	
Middle	34 (10.9)	8 (13.3)	26 (10.3)	
Distal	3 (1.0)	1 (1.7)	2 (0.8)	
Neoadjuvant treatment *n* (%)				0.531
Chemotherapy	107 (34.2)	20 (33.3)	87 (34.4)	
Chemoradiation	134 (42.8)	23 (38.3)	111 (43.9)	
Radiation	2 (0.6)	1 (1.7)	1 (0.4)	
None	70 (22.4)	16 (26.7)	54 (21.3)	
Intraoperative characteristics				
Operation type *n* (%)				0.560
MIE	115 (36.7)	24 (40)	91 (36.0)	
RAMIE	198 (63.3)	36 (60)	162 (64)	
Thoracic procedure duration median (min–range)	206 (98–406)	221 (128–340)	204 (98–406)	0.014
Thoracic procedure duration MIE (min–range)	191 (111–319)	207.5 (128–319)	187 (111–298)	0.11
Thoracic procedure duration RAMIE (min–range)	214 (98–406)	223 (169–340)	211 (98–406)	0.045
Number of lymph nodes resected (median–range)	30 (8–81)	27 (11–64)	31 (8–81)	0.084
Number of lymph nodes resected MIE (median–range)	25 (11–64)	24 (11–64)	26 (11–64)	0.586
Number of lymph nodes resected RAMIE (median–range)	32 (8–81)	28.5 (14–59)	33 (8–81)	0.112

Data are expressed as the number of cases *n* (%) or mean/median ± standard deviation (SD)

*ASA* American Society of Anaesthesiologists, *AEG* adenocarcinoma of the esophagogastric junction, *MIE* minimally invasive esophagectomy, *RAMIE* robotic-assisted minimally invasive esophagectomy

### Impact on thoracic procedure duration

The impact of thoracic width on thoracic procedure duration is shown in Table 2. Including all operations, thoracic width was associated with longer procedure duration in a univariate linear regression model (*p* = 0.014). BMI, pulmonary comorbidity, cT Stage > cT3, and preoperative radiation therapy were not associated with longer procedure duration, whereas positive cN Stage, neoadjuvant therapy and a right-sided esophagus were significantly correlated. In the multivariate linear regression analysis, only thoracic width and neoadjuvant therapy remain significant factors for prolonged thoracic procedure duration.

**Table 2:** Correlation between patient factors and thoracic procedure duration in the univariate and multivariate analysis

Univariate analysis		
Continous variables	Correlation coefficient	*p*-value
Thoracic width	− 0.139	0.014
BMI	− 0.1	0.865

Categorized variables	Median time (min)		
	No	Yes	
Pulmonary comorbidity (median)	206	207	0.811
cT stage (≥ cT3)	202	208	0.319
cN stage (*N* +)	204	214.5	0.047
Neoadjuvant therapy	195.5	210	0.013
Left-sided esophagus	214	202	0.006
Preoperative radiation therapy	204	210	0.068

Multivariate analysis	*p*-value
Thoracic width	0.026
cN stage (*N*+)	0.046
Neoadjuvant therapy	0.012
Left-sided esophagus	0.09

Univariate linear regression is used for continuous variables and Mann–Whitney *U* test for categorized variables, correlation coefficient using Spearman’s rank correlation coefficient analysis, multivariate linear regression is used for significant factors in the univariate analysis

To identify patients with small thoracic width, we tried to establish an optimal cut-off. To our knowledge, no optimal cut-off values have been reported in literature using thoracic width on vena azygos level. Seeing the trend, we performed a ROC analysis for evaluation of the thoracic cage width using pneumonia as factor. In regard to the clinical setting, we were looking for a cut-off with rather high sensitivity than specificity, since postoperative pneumonia has several possible risk factors. When defining narrow thoraces smaller than 108 mm sensitivity was 0.731 and specificity was 0.176. Sixty patients remained in the narrow thorax group (19.2%) with 41.7% female and 58.8% male patients. Comparing these two groups regarding patient and tumor characteristics, no difference was found except for higher percentage of female patients in the small thorax group and more patient with a left-sided esophagus in the wide thorax group (Table 1). Thoracic procedure duration was significantly shorter (median 204 min) in the narrow thorax group compared to the wide thorax group (median 221 min) (*p* = 0.014). Considering MIE and RAMIE procedures separately, both had longer thoracic procedure duration in the small thorax group, however, the MIE group (*n* = 115) did not reach statistical significance.

### Impact on postoperative complication rates

In the univariate logistic regression, small thoracic width had a significant correlation with higher postoperative pneumonia rates in our cohort (OR 0.97 (0.944–0.997), *p* = 0.032). Other intra- and postoperative complication rates shown in Table 3 did not show significant correlations. Comparing postoperative complications rates of small and wide thoraces according to the cut-off of 108 mm, there were significantly more anastomotic leakages registered in the small thorax group (21.7% vs. 10.3%, *p* = 0.016). More patients in the narrow thorax group suffered from postoperative pneumonia (23.3% vs. 15%), however, this difference was not statistical significant (*p* = 0.12). There was no difference regarding conversion rates, severe morbidity, wound infection, cardiovascular complications, chyle leak, readmission to ICU, as well as 30- and 90-day mortality.

**Table 3:** Intra- and postoperative complications rates of all patients, correlation between intraoperative and postoperative complication rates regarding thoracic width, comparison of narrow and wide thorax groups

Complication	All patients	Odds ratio (95% CI)	*p*-value	Narrow thorax $\le$ 107 mm	Wide thorax $\ge$ 108 mm	*p*-value
Conversion thoracic phase	8 (2.6)	0.976 (0.916–1.040)	0.455	2 (3.3)	6 (2.4)	0.671
Any morbidity	137 (43.8)	0.985 (0.965–1.005)	0.140	29 (48.8)	108 (42.7)	0.428
Severe morbidity (CDc > IIIb)	56 (17.9)	0.986 (0.961–1.013)	0.304	15 (25)	41 (16.2)	0.11
Pneumonia	52 (16.6)	0.970 (0.944–0.997)	0.032	14 (23.3)	38 (15.0)	0.120
Anastomotic leakage	39 (12.5)	0.980 (0.951–1.011)	0.207	13 (21.7)	26 (10.3)	0.016
Wound infection	8 (2.6)	1.016 (0.953–1.083)	0.624	2 (3.3)	6 (2.4)	0.671
Cardiovascular complication	27 (8.6)	1.007 (0.972–1.044)	0.692	6 (10)	21 (8.3)	0.673
Chylothorax	10 (3.2)	1.012 (0.956–1.072)	0.681	1 (1.7)	9 (3.6)	0.454
Readmission to ICU	32 (10.2)	0.978 (0.946–1.011)	0.188	8 (13.3)	24 (9.5)	0.377
30-Day mortality	6 (1.9)	1.016 (0.943–1.093)	0.679	1 (1.7)	5 (2.0)	0.875
90-Day mortality	13 (4.2)	0.978 (0.929–1.029)	0.385	3 (5.0)	10 (4.0)	0.724

Data are expressed as the number of cases *n* (%), odds ratio with confidence interval of univariate logistic regression analysis

*CI* confidence interval, *CD* Clavien–Dindo, *ICU* intensive care unit

In the multivariate logistic regression (Table 4), anastomotic leakage and thoracic cage width were significant risk factor for occurrence of postoperative pneumonia (*p* $\le$ 0.001, respectively, *p* = 0.045).

**Table 4:** Postoperative complications, multivariate logistic regression analysis

Risk factor	Pneumonia	
	Odds ratio (95% CI)	*p*-value
Pulmonary comorbidity	1.003 (0.437–2.302)	0.995
Thoracic width	0.971 (0.943–0.999)	0.045
Anastomotic leakage	4.479 (2.109–9.511)	< 0.001
Age	1.037 (0.992–1.083)	0.112
Charlson comorbidity index	0.867 (0.660–1.139)	0.306

*CI* confidence interval

## Discussion

In this study, we evaluated the impact of small thoracic width on thoracic procedure duration and postoperative complication rates in minimally invasive esophageal resection. In our European cohort, small thorax width on vena azygos level was significantly correlated with longer procedure duration in the thorax. This is the main finding in this study.

Since its implementation in 1992, MIE has nowadays become a routinely performed operation treating esophageal cancer. Avoiding the destruction of the thoracic wall, reduction of postoperative morbidity and mortality has been its main advantage [5–7, 12]. However, in an evaluation of the nationwide database in Japan from 2011 to 2012 MIE was associated with indeed less postoperative respiratory ventilation time, but with higher reoperation rates (7.0 vs. 5.3%, *p* = 0.004) [13]. Additionally, longer operation time and flat learning curves described in literature hint at a required elevated skill level and expertise of the surgeon when performing MIE [14].

Especially for surgeons in training, adequate patient selection is key for optimal training and prevention of patient impairment. Therefore, an easy preoperative evaluation of expected surgical difficulty needs to be established. Since direct evaluation of a procedures difficulty is often a subjective perception, we used procedure duration as an indicator for operation difficulty. To our knowledge, a sole report of Takeno et al. exists analyzing the utility of thoracic cage width in assessing surgical difficulty of MIE patients [15]. They found in an Asian cohort of 44 patients small area of the upper thoracic cage correlated with prolonged thoracic procedure time (*p* = 0.0119), whereas area of the lower thoracic cage did not. There was no direct correlation with postoperative morbidity rates. In our experience, the main area of interest during the thoracic part and the common position of the later anastomosis is the region where the vena azygos is crossing the esophagus sagittally. We consequently defined the horizontal distance from thoracic wall to the esophagus in this region as thoracic width in our study. Additionally, in clinical practice the measurement of one distance seems more practicable than measuring an area. In comparison to Takeno et al., we could examine a large European cohort consisting of 313 patients including 198 operations with robotic assistance.

We found in the univariate analysis as well as in the multivariate analysis correlation between narrow thoraces and long thoracic procedure duration (*p* = 0.014, respectively, 0.006). When creating a cut-off for quite narrow thoraces (≤ 107 mm), the same effect was found. It remains unclear which part of the procedure is more time consuming in patients with narrow thoraces. The higher rate of anastomotic leakages in the narrow thorax group might suggest that creation of the circular stapled anastomosis is more demanding with limited space, rather than the dissecting part of the operation. Finally, to answer this question exact documentation of the required procedure times of all parts of the operation is necessary.

An esophagus located on the left side of the trachea in the upper mediastinum was also associated with shorter procedure duration in the univariate analysis. Interestingly, in contrast to the Asian cohort of Yoshida et al. only 2.9% of our patients had a completely deep seated esophagus (vs. 30.4% in the Asian cohort) [11]. In their study, a deep seated esophagus had a strong positive influence on the difficulty of thoracoscopic esophagectomy. In our European cohort, a completely deep seated esophagus is not as common as in Asian patients. Furthermore, even patients with a left-sided esophagus did not increase surgical difficulty. One could argue that these patients frequently have a wide thorax in our cohort and thus offer more space during procedures.

In the end, anatomic conditions seem to differ greatly between Asian and European cohorts regarding thorax size and esophageal location. Thus, European surgons might not be trained enough for surgical difficulties arising when operating in small thoraces. This factor should always be taken into consideration when comparing procedure times and surgical difficulty. In addition, special anatomic circumstances, such as chest wall defomities, including chest wall dystrophy or pectus excavatum, should always be taken into account for increased surgical difficulty due to small thoracic cavities and narrow intercostal spaces.

Interestingly, also application of neoadjuvant therapy was significantly correlated with longer procedure time in both analysis. In our experience, this might be caused by an extended preparation due to an advanced underlying disease, as well as possible structural changes of the tissue after radiation. Preoperative radiation therapy alone did not reach statistical significant correlation with prolonged procedure duration. There was no correlation between neoadjuvant treatment and thoracic cage width.

Comparing small and wide thorax groups in MIE and RAMIE patients separately, a significant duration difference can be shown for the RAMIE patients (223 min vs. 211 min, *p* = 0.045). However, MIE patients had the same tendencies, just did not reach statistical significance (*n* = 115, 207.5 min vs. 187 min, *p* = 0.104). In order to eliminate eventual bias by the learning curve for robotic-assisted procedures we excluded the first 70 RAMIE. Even without these operations the correlation between thoracic width and thoracic procedure time is significant (*p* = 0.038, not shown in the table). Regarding postoperative complication rates, we found significant correlation between pneumonia and small thoracic width in the univariate analysis (*p* = 0.032). This might not be directly associated but could be caused by the extended operation time and the consecutive extended ventilation time. Comparing both groups, pneumonia rates had a trend to be higher in the narrow thorax group without reaching significance, however, we experienced significantly more anastomotic leakages in the small thoracic group (13 vs. 26, *p* = 0.016). Although we could not confirm this result in the logistic regression, increased rate of anastomotic leakages might be caused by hampering of free movement in narrow thoraces, which is crucial for creation of a well vascularized and tension-free anastomosis. Still, risk factors for anastomotic leakages after MIE are variant and controversially discussed; for definite conclusions, the influence of thoracic width needs be addressed in further studies.

Limitations of this study clearly include the single-center design. In order to create an evidence-based recommendation, a randomized comparison between experienced surgeon and trainee would be correct, which is ethically not easily practicable. Additionally, procedure duration can only be a hint for estimation of surgical difficulty—for direct evaluation of surgical difficulty and patient safety, a standardized postoperative questionnaire filled out by the surgeon or trainee would be ideal.

Considering these findings, patients with narrow thoracic width on the level of the vena azygos (≤ 107 mm) should be preferably operated by surgeon with expertise in MIE.

## Conclusion

In this study, narrow thoracic width on crossing of vena azygos level is associated with prolonged thoracic procedure duration and higher postoperative pneumonia rates in a European single-center institution. For patients’ safety and optimal learning effect, patients with narrow thoraces should be preferably operated by surgeons with expertise in minimally invasive esophagectomy.
